# Genetic Architecture of Early Vigor Traits in Wild Soybean

**DOI:** 10.3390/ijms21093105

**Published:** 2020-04-28

**Authors:** Janice Kofsky, Hengyou Zhang, Bao-Hua Song

**Affiliations:** Department of Biological Sciences, University of North Carolina at Charlotte, Charlotte, NC 28223, USA; jkofsky@uncc.edu (J.K.); hengyou.zhang@gmail.com (H.Z.)

**Keywords:** *Glycine soja*, wild soybean, early vigor, early growth, genome-wide association study, GWAS

## Abstract

A worldwide food shortage has been projected as a result of the current increase in global population and climate change. In order to provide sufficient food to feed more people, we must develop crops that can produce higher yields. Plant early vigor traits, early growth rate (EGR), early plant height (EPH), inter-node length, and node count are important traits that are related to crop yield. *Glycine soja*, the wild counterpart to cultivated soybean, *Glycine max*, harbors much higher genetic diversity and can grow in diverse environments. It can also cross easily with cultivated soybean. Thus, it holds a great potential in developing soybean cultivars with beneficial agronomic traits. In this study, we used 225 wild soybean accessions originally from diverse environments across its geographic distribution in East Asia. We quantified the natural variation of several early vigor traits, investigated the relationships among them, and dissected the genetic basis of these traits by applying a Genome-Wide Association Study (GWAS) with genome-wide single nucleotide polymorphism (SNP) data. Our results showed positive correlation between all early vigor traits studied. A total of 12 SNPs significantly associated with EPH were identified with 4 shared with EGR. We also identified two candidate genes, *Glyma.07G055800.1* and *Glyma.07G055900.1*, playing important roles in influencing trait variation in both EGR and EPH in *G. soja*.

## 1. Introduction

The cultivated soybean, *Glycine max*, is an important legume crop that supplies the majority of protein meal and oilseed worldwide [1]. Crop improvement is a continuous necessity as the demands in agriculture increase due to a changing environment and rising population. Modern soybean crops are challenged by abiotic and biotic stress. In order for soybean production to keep up with the growing population, novel modifications must be made to the current crop beyond modern breeding practices [2]. The wild counterpart to the cultivated soybean, *Glycine soja*, is analyzed for genetic associations to early vigor traits that are not present in the cultivated population.

*G. max* diverged from *G. soja* 0.27 [3] or 0.8 million years ago (mya) [4], leaving much of the wild genetic variability behind [5]. The cultivated soybean further diverged as a result of domestication in China 6000–9000 years ago; however, the wild soybean continues to inhabit a wide range of areas across Eastern Asia [3,6]. The gene pool in wild soybean retains the genes and alleles lost during the process of artificial breeding practices and, more importantly, the initial domestication of the soybean, which contributes to 16.2% and 31% reduction in genetic diversity, respectively [7]. Selection and improvement within *G. max* have been shown to have a significant effect on diversity on all twenty chromosomes [8,9]. By exploiting the genetic diversity harbored in the wild soybean, we are able to discover novel sources of agronomical superiority.

Early vigor is a combination of traits that impact the eventual success and yield of a plant. The early vigor phenotypic traits studied in *G. soja* are node count, inter-node length, early growth rate, and early plant height. Early growth rate (EGR) and early plant height (EPH) are further analyzed for genotypic dissection using genome-wide association studies (GWAS). These traits have not been studied or used for genetic association in wild soybean thus far. The node count and inter-node length are representative of the density of plant foliage, as each node results in a branching compound leaf. There is a significant correlation between the yield per soybean plant and branch number [10]. Measurements of mature plant height in wild soybeans are relatively challenging due to its fragile stem and vining nature. Therefore, EPH can be used as an analysis of height while the plant is still in early growth stages. EGR is an important complex trait, and often associated with the ultimate success of the plant. Seed vigor is determined partially by an evaluation of seedling growth [11], which was initially established in the 1950s to represent the success and productivity of the resulting plant [12]. All traits analyzed are representative of plant success and a measure of vigor.

A genome-wide association study (GWAS) was used in this study to determine genetic associations with complex agronomically beneficial traits, EPH and EGR. GWAS has significant advantages over the tradition approach of complex candidate loci discovery, quantitative trait loci (QTL) mapping. QTL mapping relies on a crossed mapping population from only two individuals to contain all genetic diversity for the trait being analyzed. In addition, QTL mapping is limited by its resolution in the mapping population due to limited recombination events [13]. Although first demonstrated for use in human disease [14], GWAS has been widely applied to plants for over a decade to identify genes responsible for quantitative traits. While the QTL mapping approach uses just two genotypes for genetic variation discovery, GWAS utilizes an unlimited sample size and takes advantage of the genetic and phenotypic diversity contained in the extensive natural population and thus providing higher mapping resolution than bi-parental populations [15,16]. The wild soybean is an ideal system for GWAS, as it can be maintained by self-fertilization, allowing for repeated screening of genetically similar individuals. GWAS has previously been used to dissect phenotypic traits related to biotic stress resistance [17,18,19], abiotic stress tolerance [20], and seed composition [21,22] in wild soybean.

The value of crop wild relatives in crop improvement has been progressively recognized in the past decades [23]. The wild soybean, *G. soja*, has been used in the genetic dissection of soybean growth [24,25], yield [26], seed composition [21,27], abiotic stress tolerance [20,28,29,30,31,32], and biotic stress resistance [18,33,34,35,36,37,38,39,40,41,42]. However, to our knowledge, using *G. soja* to study early vigor phenotypic traits, such as early growth rate, early plant height, node count, and inter-node length, has never been reported. To explore the genetic diversity harbored in the wild soybean, we used GWAS to effectively characterize the genetic architecture of early vigor traits. This study aims to make use of a cutting-edge quantitative trait loci discovery method to dissect the genetic basis of agronomically beneficial traits, EGR and EPH, and their relationships to inter-node length and node count to further crop-improvement techniques.

## 2. Results

### 2.1. Correlation in Phenotypic Traits

All trait relationships studied on 225 ecotypes of *G. soja,* inter-node length to node count, inter-node length to EGR, inter-node length to EPH, node count to EPH, node count to EGR, and EGR to EPH, expressed significant positive correlations, *p* < 0.001, by Spearman’s correlation test (Table 1). As a trend, plants with higher growth rate and height had more nodes and the distance between each of the nodes was greater.

### 2.2. Genome-Wide Association Analysis

**Figure 1:**
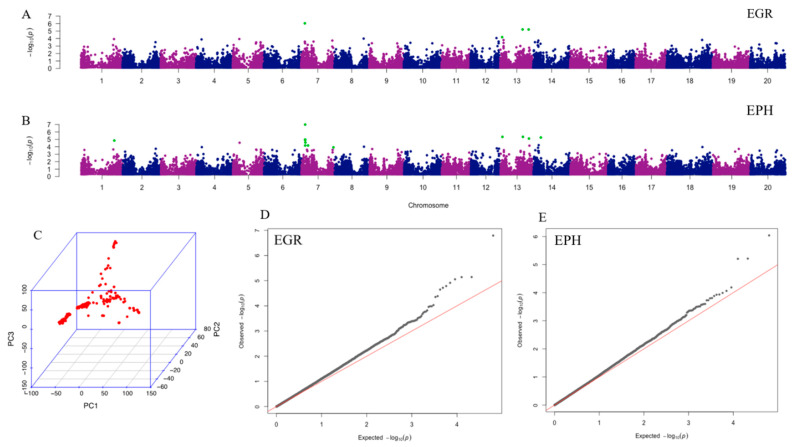
Results of principle component analysis (PCA) and genome-wide association analysis (GWAS). Manhattan plots illustrate the GWAS results of EGR (**A**) and EPH (**B**). X-axis represents single nucleotide polymorphism (SNP) positions across the entire genome by chromosome and the y-axis is the negative logarithm *p*-value: -log10 (*p*) of each SNP. Significant SNPs with chromosome-wide false discovery rate (FDR) adjustment are highlighted in green. The corresponding QQ plots for EGR and EPH are shown in (**D**) and (**E**), respectively. For Q-Q plots, x-axis represents expected −log10 (*p*) and y-axis is observed −log10 (*p*) of each SNPs. (**C**) Plot of PCA result with all 225 ecotypes. EGR: early growth rate; EPH: early plant height.

A total of 31,726 filtered single nucleotide polymorphisms (SNPs) was used for the principle component analysis (PCA) and GWAS. The PCA result was shown in Figure 1C. The Mixed Linear Model (MLM) combined with kinship and structure control resulted in four significant SNPs associated with EGR and 12 significant SNPs associated with EPH, with chromosome-wide false discovery rate (FDR)-adjusted *p* < 0.05 (Table 2). All significant SNPs associated with EGR are also significant for EPH. The most highly associated SNP to EGR and EPH, ss715598271, is significant (*p* < 0.05) by all *p*-value adjustment and threshold tests (chromosome-wide Bonferroni, genome-wide Bonferroni, and genome-wide FDR). SNP marker ss715598271 explains 10.85% of EGR phenotype variation and 12.42% of EPH phenotype variation. Bracketing markers to ss715598271, ss715598270, and ss715598272 are also shown to be significant by chromosome-wide Bonferroni threshold adjustment in EPH, explaining 8.34% and 8.17% of the phenotypic variation, respectively. Manhattan plots of EGR and EPH (Figure 1A,B) visualize all significant SNPs by chromosome, with corresponding Q-Q plots (Figure 1D,E). The heritability was estimated to be 0.64 for EPH and 0.71 for EGR.

### 2.3. In-Depth Candidate Loci Investigation

The SNP ss715598271, which is significantly associated with both EGR and EPH, and its bracketing markers (ss715598269, ss715598270, and ss715598272) associated with EPH, co-localize with a previously identified large QTL, plant height 19-5 [43]. In addition, these four SNPs are within 50 kb adjacent to SNP ss715598304, an intergenic marker significantly associated with EPH, which is located under the known QTLs: plant height 3-3 [45], plant height 25-6 [46], and plant height 19-5 [43] (Table 2). The SNP marker ss715614175 on chromosome 13, significantly associated with both EGR and EPH, located within the previously-described QTL related to plant height: Plant Height 26-11 [44] (Table 2).

**Figure 2:**
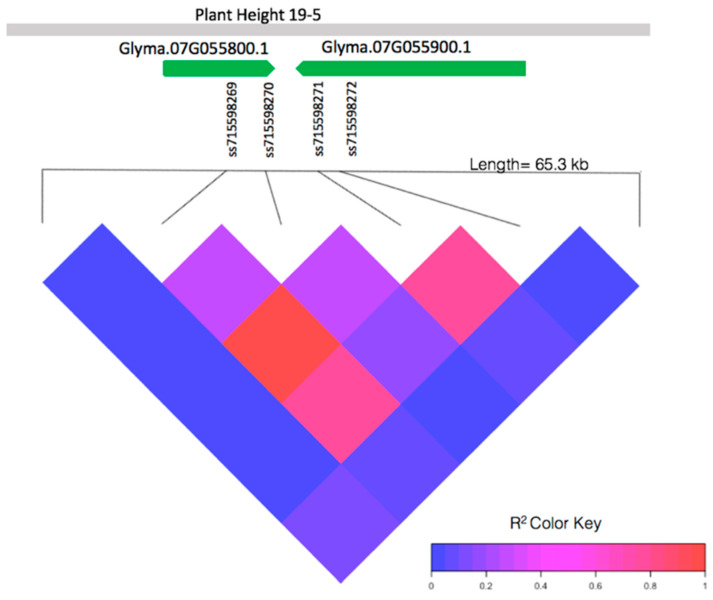
Pairwise linkage disequilibrium (LD) between SNPs in the local genes of interest: *Glyma.07G055800.1*, *Glyma.07G055900.1*, and the known QTL, plant height 19-5 [43], in relation to SNPs ss715598269, ss715598270, ss715598272 (significantly associated with EPH), and ss715598271 (significantly associated with both EPH and EGR).

The pairwise linkage disequilibrium (LD) analysis of a 65.3-kb window containing the four significant SNPs (ss715598269, ss715598270, ss715598271, and ss715598272) from 4,915,929 bp to 4,928,272 bp on chromosome 7 shows high linkage within this region (Figure 2). Two markers, ss715598269 and ss715598270, are within the intron and 3′ untranslated region of gene *Glyma.07G055800.1*, respectively. Two markers, ss715598271 and ss715598272, are within the intron of gene *Glyma.07G055900.1*. No significant LD is found near the other significant SNPs. These, therefore, were not investigated further for candidate genes.

There is a significant association between ss715598271 allele polymorphism (A/C) and studied traits EPH and EGR. The A allele morph is associated with higher EGR and higher EPH than the C allele morph at this marker (*p* < 0.02) (Figure S1A,B). The significant association between allele and trait suggests that ss715598271 might be located in a region crucial for gene functioning.

*Glyma.07G055800.1* and *Glyma.07G055900.1* are promising candidate genes associated with both EPH and EGR. *Glyma.07G055800.1* is predicted to encode a transmembrane protein containing DoH and Cytochrome b-561/ferric reductase transmembrane domains [47]. Its closest homolog in *Arabidopsis* is Cytochrome b561/ferric reductase transmembrane with DOMON-related domain protein (CYB561). This protein is responsible for catalyzing transmembrane electron transfer by ascorbate, which is a key metabolite in growth and development of plants [48]. *Glyma.07G055900.1* is predicted to encode a Tetratricopeptide repeat (TPR)-like super family protein, playing a role in protein binding and translation initiation [47].

## 3. Discussion

Significant correlations were found between all traits studied, suggesting that EPH, EGR, inter-node length, and node count are all related phenotypic traits (Table 1). The positive relationship between inter-node length and node count, inter-node length and EPH, and internode-length and EGR demonstrates the combined effect of elongation of the inter-nodes along with the addition of new nodes in early growth of the wild soybean. Each node of the wild soybean is accompanied by one branching compound leaf. Therefore, the observed increase in node count with EGR and EPH relates directly to foliage increase and acquisition of available light, having a cumulative effect on growth. The total yield has previously been shown to be positively correlated with the branching of a soybean [10]. A positive correlation between all early vigor traits tested demonstrates that the traits chosen are reliable indicators of early vigor and eventual success of a plant.

We identified a total of 12 significant SNPs for EPH with 4 shared significant SNPs for EPH and EGR. The most compelling results were found on chromosome 7, in which a contiguous set of markers, ss715598269, ss715598270, ss715598271, and ss715598272, are highly significant, revealing the importance of this genomic location to EGR and EPH. This locus also colocalize with a known QTL, plant height 19-5 [43], and is adjacent to two QTLs, plant height 3-3 [45] and plant height 25-6 [46]. Although these consistently mapping QTLs were previously identified, variation in this region might be unique to *G. soja* as previously described in the finding of a *G. soja*-unique salt-tolerant gene *GmCHX1* [49]. Further analysis showed that significant LD is evident in this region. High LD can suggest that this genomic region might have been under positive selection in its natural environment, which is expected as plant height is an ecologically important trait. We were able to determine an allelic correlation with EPH and EGR at marker ss715598271. The A allele morph is correlated with higher EGR and higher EPH than the C allele morph at this marker. This finding suggests that this marker might be linked to genes playing important roles in influencing these trait variations in *G. soja*. This is supported by significant surrounding markers and high LD in this region.

Two candidate genes, *Glyma.07G055800.1* and *Glyma.07G055900.1* on chromosome 7, were identified as significantly related to EPH and EGR. Significant markers are located within the introns of these genes and the untranslated 3′ region of *Glyma.07G055800.1*. *Glyma.07G055800.1* encodes a Cytochrome *b561* transmembrane protein, or CYB561 in Arabidopsis, a transmembrane protein involved in electron transport [50]. *Glyma.07G055900.1*, encodes a Tetratricopeptide repeat-like super family protein, homologous to *Reduced Chloroplast Coverage 1* (*REC1*) in Arabidopsis. The Tetratrichopeptide (TPR) domain protein has been shown to be involved in plant height-related phenotypes in Maize and Arabidopsis [51,52] and is a known motif in plant hormone signaling responses, such as auxin, gibberellin, and cytokinin [53,54]. It is not unlikely that multiple individuals of this protein family share a similar influence on traits in various locations in the genome. It has been suggested that *REC1* is involved in establishing and maintaining chloroplast coverage in Arabidopsis and could be manipulated in order to influence energy intake and yield [55]. *G. soja*-type *REC1, Glyma.07G055900.1*, is likely involved in similar chloroplast coverage and plant hormone signaling pathways to those found in Maize and Arabidopsis, with a direct relationship to photosynthesis efficiency and growth rate, and therefore is a promising candidate gene to further investigate for crop improvement.

This study identified two candidate genes, *Glyma.07G055800.1* and *Glyma.07G055900.1,* related to early vigor traits, EGR and EPH. These traits are highly beneficial to agriculture and might have been artificially selected for in breeding practices within cultivated soybean *G. max.* However, the extent of adaptation by selection in the cultivated soybean population is limited by the small gene pool. We explored the genetic architecture of these traits in the more diverse wild soybean *G. soja* to further improve our cultivation practices beyond the limited genotype of *G. max*. Further investigation, such as function validation of these candidate genes, will shed light on understanding the molecular mechanisms of these agriculturally and ecologically important traits. Meanwhile, it will provide a foundation for soybean trait improvement by traditional breeding, biotechnology, or genome editing. The long-term goal is to make full use of the wild genetic resources to meet the global challenges of agriculture sustainability and food security.

## 4. Materials and Methods

### 4.1. Plant Materials and Phenotyping

**Figure 3:**
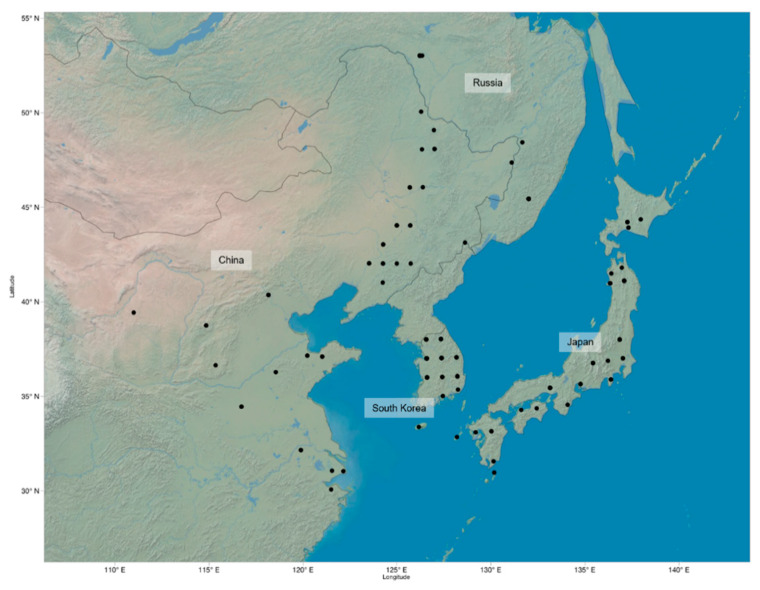
Geographic distribution of known locations of 225 *G. soja* accession: Each point marks a geographic location where the samples were originally collected.

In total, 225 *G. soja* accessions obtained from the USDA Soybean Germplasm Collection were used for measurements and analysis. The original geographic distribution of these accessions includes areas in China, Japan, Russia, and South Korea (Figure 3). All seeds were manually scarified and germinated on filter paper for three days, after which 4–5 seedlings per genotype were transplanted into Miracle-Gro soil in separate cells of a 3 × 5 growing tray. Optimal growing conditions were kept constant at 27 °C and 12 h light/day in the greenhouse at University of North Carolina at Charlotte (NC, USA). The plants were watered regularly to keep soil moist.

Four phenotypic traits were recorded or measured with a caliper to the nearest 0.1 mm on each accession: node count, internode length, EGR, and EPH. EPH was measured at 20 days after germination, and node count, EGR, and internode length were obtained as the average of three recordings taken at days 7, 14, and 20 after germination. For each accession, measurements from two or three seedlings, quality filtered by coefficient of variance and noted for damage during growth, were averaged for each trait used in the analysis (Table S1).

### 4.2. Genotypic Data

Previously identified markers, single nucleotide polymorphisms (SNPs), for all 225 *G. soja* accessions were obtained from SoyBase (http://soybase.org) [47]. All markers were originally determined by the use of the Illumina Infinium SoySNP50K iSelect BeadChip, with 52,041 total verified SNP markers [56]. All markers with a minor allele frequency (MAF) <0.05 or missing rate of >10% were filtered out of the analyzed data, leaving 31,726 SNPs. All cleaned data were imputed using BEAGLE (v 3.3.1) [57,58,59].

### 4.3. Phenotype Analysis

Pair-wise correlations among the four traits were evaluated with Spearman’s analysis due to non-normal distributions to determine the relationship between traits (specifically, the relationship between height and growth traits with the number and frequency of nodes and, therefore, relative foliage quantity). It has been suggested that normalization is not necessary for genome-wide association analysis when the data size is relatively large to avoid error, such as false positives [60]. Thus, the phenotype data was not normalized in this study. The heritability of the quantitative traits was estimated using the following equation: (additive genetic variance)/(additive genetic variance + error variance) [61].

### 4.4. Genome-Wide Association Analysis

A PCA was conducted using the GAPIT package [62]. A Mixed Linear Model (MLM) in TASSEL [63] was performed to analyze the associations of EGR and EPH with SNPs for all 225 accessions. A principle component (PC) value of three, selected by analysis of quantile-quantile plots at various PC values, and a scaled IBS Kinship matrix was used to control for the population structure. Multiple significance threshold tests were used in order to substantiate the determination of significant SNPs. The significance threshold was determined by chromosome-wide false discovery rate (FDR) resulting in an adjusted *p*-value (*q*-value) for each marker [64]. Genome-wide FDR, along with genome-wide and chromosome-wide Bonferroni adjustments [65,66], were also used to validate the significant SNPs at *p* < 0.05 or *q* < 0.05 cutoff.

### 4.5. Investigation of Candidate Loci

All genes within 50 kb of significant SNPs were evaluated for potential association with each phenotype. The interval was used based on the average linkage disequilibrium reported in *G. soja*. The annotated soybean reference genome, Wm82.a2.v1 (SoyBase, http://soybase.org), was used to determine these genes along with further investigation into the function using Phytozome [67], TAIR [68], and BLAST2GO [69]. Known QTLs for each phenotype from SoyBase [47] in each candidate loci were considered for validation of candidate genes. SNP allele to phenotype comparison was done by nonparametric median tests. Pairwise linkage disequilibrium (LD) was calculated with TASSEL and visualized with the LDheatmap R package [70].

## Figures and Tables

**Table 1:** Phenotypic variation and correlations of studied early vigor traits in wild soybean: Mean, standard deviation (StDev), and coefficient of variance (CV). The units of early plant height (EPH) and inter-node length are mm. Positive significant correlation between traits: early growth rate (EGR), EPH, node count, and inter-node length by Spearman’s ρ correlation coefficient.

Trait	Phenotypic Variation			Positive Significant Correlation between Traits		
	Mean	StDev	CV	EPH	Node Count	Inter-Node Length
EGR	16.02	8.34	52.05	0.9861	0.7023	0.8674
EPH	244.68	127.2	51.99	-	0.6911	0.8838
Node Count	4.34	0.93	21.33	-	-	0.3449
Inter-node Length	54.55	24.97	45.76	-	-	-

**Table 2:** Significant SNP markers associated with early growth rate (EGR) and early plant height (EPH): The q-value given is the chromosome-wide FDR-adjusted *p*-value. Significance in CB (Chromosome-wide Bonferroni threshold), GB (Genome-wide Bonferroni threshold), and GFDR (Genome-wide FDR adjustment) with *p* < 0.05 are indicated with (*). The position is physical position on the chromosome (Chr.) in base pair.

SNP		Chr	Position	*r* ^2^	Location	Associated Gene	Associated QTL	*p*-value	*q*-value	CB	GB	GFDR
**EGR**	ss715598271	7	4924020	0.1085	Intron	*Glyma.07G055900.1*	*Plant Height 19-5* [43]	9.14E-07	0.001431	*	*	*
	ss715614175	13	19487316	0.071	Intergenic	*-*	Plant Height 26-11 [44]	6.50E-05	0.042055	-	-	-
	ss715615103	13	31173270	0.0911	Intergenic	*-*		6.12E-06	0.006046	*	-	-
	ss715616082	13	39280839	0.0944	5UTR	*Glyma.13G292800.1*		6.23E-06	0.006046	*	-	-
**EPH**	ss715579500	1	45269059	0.0792	Intergenic	*-*		2.26E-05	0.028024	*	-	-
	ss715598269	7	4915929	0.0682	Intron	*Glyma.07G055800.1*	Plant Height 19-5 [43]	1.04E-04	0.027144	-	-	-
	ss715598270	7	4918294	0.0834	3UTR	*Glyma.07G055800.1*	Plant Height 19-5 [43]	1.64E-05	0.010022	*	-	-
	ss715598271	7	4924020	0.1242	Intron	*Glyma.07G055900.1*	Plant Height 19-5 [43]	1.62E-07	0.000254	*	*	*
	ss715598272	7	4928272	0.0817	Intron	*Glyma.07G055900.1*	Plant Height 19-5 [43]	1.92E-05	0.010022	*	-	-
	ss715598304	7	5214440	0.0816	Intergenic	*-*	Plant Height 19-5 [43] Plant Height 3-3 [45] Plant Height 25-6 [46]	4.11E-05	0.016091	-	-	-
	ss715598895	7	8788505	0.0668	Intron	*Glyma.07G094100.1*		1.04E-04	0.027144	-	-	-
	ss715598145	7	42926704	0.0628	CDS	*Glyma.07G251700.1*		1.86E-04	0.041611	-	-	-
	ss715614175	13	19487316	0.09	Intergenic	*-*	Plant Height 26-11 [44]	7.24E-06	0.007026	*	-	-
	ss715615103	13	31173270	0.0893	Intergenic	*-*		7.18E-06	0.007026	*	-	-
	ss715616082	13	39280839	0.0874	5UTR	*Glyma.13G292800.1*		1.22E-05	0.007893	*	-	-
	ss715620138	14	9595999	0.0873	Intergenic	-		8.84E-06	0.013826	*	-	-

## Supplementary Materials

The following are available online at https://www.mdpi.com/1422-0067/21/9/3105/s1.
