# Supplementary material for: Genetic Architecture of Early Vigor Traits in Wild Soybean
Source: Int J Mol Sci. 2020 Apr 28;21(9):3105. doi: 10.3390/ijms21093105 (PMC7247153; doi:10.3390/ijms21093105)
Supplement: Supplementary file 1 [file ijms-21-03105-s001.pdf]

Table S1. Phenotypes measured and calculated in this study and geographic origin of the samples. Phenotypes include early plant height, early growth rate, and inter-node length, and node count.

Accession	Country	Early Plant Height (mm)	Early Growth Rate (mm/day)	Inter-node length (mm)	Node Count
**PI101404A**	China	325.83	21.07	58.71	5
**PI101404B**	China	46.95	1.59	87.42	3
**PI163453**	China	384.6	26.68	69.05	6
**PI407288**	China	323.77	21.25	94.9	5
**PI407296**	China	458.9	26.49	47.28	5
**PI407298**	China	304.94	19.19	71.34	3
**PI407302**	China	261	17.64	93.06	4
**PI407303**	China	363.27	25.21	56.18	6
**PI407304**	China	290.86	18.86	54.77	5
**PI407307**	China	326	23.33	29.06	5
**PI458537A**	China	183.52	12.21	44.01	5
**PI458540D**	China	291.02	22.16	50.7	5
**PI464866A**	China	168.31	10.27	80.73	5
**PI464889B**	China	99.84	6.41	10.44	4
**PI464889C**	China	209.03	12.06	77.4	3
**PI464890A**	China	326.77	22.34	44.94	5
**PI464891B**	China	300.21	18.27	21.53	5
**PI464925C**	China	250.03	15.66	6.01	4
**PI464926**	China	251.98	17.59	34.21	5
**PI464927A**	China	164.56	10.35	20.9	6
**PI464927B**	China	202.73	13.99	37.4	5
**PI464928**	China	203.03	12.87	31.88	5
**PI468396B**	China	62.7	3.91	29.92	3
**PI468397A**	China	125.45	8.33	50.4	4

**PI468398B**	China	18.04	1.04	62.51	3
**PI468399B**	China	100.48	6.72	64.33	5
**PI483466**	China	205.39	13.63	40.78	5
**PI483467**	China	383.69	24.39	9.9	5
**PI483468A**	China	281.89	18.17	22.21	5
**PI522179**	China	116.06	7.07	42.9	4
**PI522180**	China	90.83	4.97	51.4	4
**PI522182A**	China	212.79	15.99	42.19	5
**PI522182B**	China	487.47	31.88	37.03	5
**PI532450**	China	344.47	22.78	48	5
**PI532453A**	China	257.09	17.84	67.53	5
**PI549037**	China	282.56	20.04	86.52	5
**PI549047**	China	515.87	32.14	58.39	5
**PI549048**	China	116.63	6.51	75.46	4
**PI597458B**	China	516.23	34.49	11.66	6
**PI597461B**	China	477.87	30.72	87.11	6
**PI464936B**	China	294.19	20.43	53.49	6
**PI464937A**	China	170	10.27	43.51	5
**PI464938**	China	174.55	11.9	43.44	5
**PI468918**	China	209.73	12.93	61.27	5
**PI479746B**	China	387	26.03	62.71	5
**PI479749**	China	34.8	1.61	28.52	3
**PI479750**	China	322.9	22.78	33.66	4
**PI479751**	China	202.8	13.22	58.2	4
**PI366121**	Japan	124.25	7.78	33.64	4
**PI366122**	Japan	258.51	16.91	44.87	4
**PI366123**	Japan	251.4	18.24	88.54	4
**PI366124**	Japan	349.77	22.27	89.77	5

**PI378684A**	Japan	358.2	22.8	66.41	5
**PI378690**	Japan	380.3	27.36	34.35	5
**PI378691**	Japan	76.95	5.51	36.19	2
**PI378697A**	Japan	339.55	19.51	26.92	5
**PI378698**	Japan	272.48	17.69	103.17	5
**PI378699A**	Japan	382.6	25.56	49.54	6
**PI378701A**	Japan	490.27	25.58	56.51	6
**PI406684**	Japan	292.64	15.84	57.13	4
**PI407030**	Japan	223.98	14.64	68.89	5
**PI407034**	Japan	217.86	11.93	39.73	4
**PI407037**	Japan	344.97	23.54	58.55	4
**PI407038**	Japan	158.6	11.25	90.55	6
**PI407044**	Japan	295.88	18.64	85.25	5
**PI407050**	Japan	337.2	18.42	49.39	4
**PI407052**	Japan	214.63	14.84	70.59	4
**PI407053**	Japan	186.25	13.36	101.32	3
**PI407077**	Japan	135.85	9.11	59.25	4
**PI407083**	Japan	96.69	7.09	70.66	4
**PI407089**	Japan	281.52	17.14	81.08	5
**PI407097**	Japan	232.55	15.83	37.35	4
**PI407120**	Japan	310.52	20.82	91.14	4
**PI407124**	Japan	135.79	9.28	91.4	4
**PI407144**	Japan	126.57	6.56	47.29	3
**PI407157**	Japan	239.85	15.94	24.77	5
**PI407286**	Japan	247.9	16.98	55.99	4
**PI486220**	Japan	111.82	6.58	8.91	3
**PI487428**	Japan	181.3	9.4	21.43	4
**PI487430**	Japan	260.13	16.37	39.41	4

**PI487431**	Japan	131.39	7.6	74.15	3
**PI504287A**	Japan	452.35	25.29	27.05	4
**PI507582**	Japan	303.14	16.69	64.51	3
**PI507609**	Japan	356.3	24.15	49.45	2
**PI507615**	Japan	453.31	30.15	35.22	6
**PI507619B**	Japan	257.77	18.67	52.35	4
**PI507632**	Japan	454.69	29.71	55.19	6
**PI507638**	Japan	304.63	21.43	40.33	5
**PI507644**	Japan	247.16	16.77	95.13	4
**PI507656**	Japan	266.5	18.79	65.11	5
**PI507667**	Japan	239.22	14.17	67.48	4
**PI508066**	Japan	194.8	10.42	46.25	5
**PI508067**	Japan	275.28	18.58	78.06	5
**PI508069**	Japan	286	18.18	36.75	5
**PI378695A**	Japan	162.6	11.92	8.39	6
**PI378696A**	Japan	243.11	14.52	16.75	5
**PI339732**	South Korea	310.63	21.54	63.24	6
**PI339871A**	South Korea	40.8	2.41	91.1	4
**PI407167**	South Korea	89.11	5.56	7.75	4
**PI407174**	South Korea	134.74	8.57	61.29	4
**PI407183**	South Korea	390.3	24.67	58.99	5
**PI407184**	South Korea	154.18	9.49	38.96	3
**PI407190**	South Korea	127.75	7.99	69.31	5
**PI407195**	South Korea	511.27	30.41	53.15	5
**PI407198**	South Korea	227.34	14.93	70.08	5
**PI407200**	South Korea	27.73	0.84	71.44	3
**PI407201**	South Korea	222.97	13.88	68.55	4
**PI407202**	South Korea	351.18	26.75	77.72	5

**PI407209**	South Korea	122.03	7.75	89.06	4
**PI407217**	South Korea	38.53	2.11	31.38	3
**PI407220**	South Korea	41.17	2.08	69.23	2
**PI407221**	South Korea	66.7	3.52	50.15	4
**PI407229**	South Korea	36.98	1.03	69.01	3
**PI407235**	South Korea	87.05	5.05	45.03	3
**PI407240**	South Korea	79.21	5.06	38.43	4
**PI407246**	South Korea	121.92	6.39	77.38	3
**PI407248**	South Korea	37.85	2.39	46.07	3
**PI407249**	South Korea	110.15	6.61	71.47	5
**PI407254**	South Korea	23.18	0.59	87.69	3
**PI407262**	South Korea	36.7	2.09	65.28	4
**PI407270**	South Korea	155.03	10.38	52.92	4
**PI407271**	South Korea	101.68	5.63	87.96	4
**PI407275**	South Korea	117.03	6.97	36.08	4
**PI407278**	South Korea	232.25	16.7	43.83	5
**PI407308**	South Korea	349.97	22.31	49.66	5
**PI407315**	South Korea	149.75	8.92	42.37	5
**PI407318A**	South Korea	148.57	10.39	75.4	4
**PI407322**	South Korea	27.11	1.36	55.38	2
**PI424008A**	South Korea	293	19.17	53.3	5
**PI424014**	South Korea	221.97	16.01	61.79	2
**PI424025B**	South Korea	121	5.96	65.28	3
**PI424032**	South Korea	220.74	15.8	82.67	4
**PI424059B**	South Korea	279.22	18.31	70.3	5
**PI424063**	South Korea	152.62	9.87	75.55	4
**PI424064**	South Korea	280.22	17.71	178.15	6
**PI424079**	South Korea	322.57	22.27	113.68	5

**PI424082**	South Korea	108.2	7.12	113.09	4
**PI424088**	South Korea	321.32	21.46	52.56	4
**PI424093**	South Korea	157.63	10.79	65.03	4
**PI424096**	South Korea	71.42	3.05	49.45	3
**PI424102A**	South Korea	26.72	0.54	33.55	3
**PI424117**	South Korea	96.23	5.47	56.38	4
**PI424121**	South Korea	36.3	1.38	76.74	4
**PI562531**	South Korea	72.58	4.49	48.62	4
**PI562544**	South Korea	16.78	1.17	27.1	2
**PI562550**	South Korea	217.17	15.23	32.98	6
**PI562551**	South Korea	148.83	9.76	76.06	4
**PI562557**	South Korea	420.6	27.34	79.6	6
**PI562558**	South Korea	478.77	31.06	65.58	5
**PI562568**	South Korea	501.7	35.09	68.56	6
**PI407279**	South Korea	37.56	1.74	103.32	3
**PI407281**	South Korea	134.33	8.02	72.09	4
**PI342621C**	Russia	197.15	12.88	18.65	4
**PI423990A**	Russia	442.1	27.47	77.04	6
**PI423993**	Russia	120.55	6.93	97.84	3
**PI423995**	Russia	308.85	21.14	95.09	6
**PI423996**	Russia	233.1	14.17	61.29	4
**PI423997**	Russia	201.13	13.03	71.73	5
**PI423999B**	Russia	244.27	16.07	74.39	5
**PI424000**	Russia	350.55	23.97	50.65	6
**PI424001**	Russia	229.7	15.61	28.29	4
**PI424002**	Russia	224.93	14.46	59.81	3
**PI507722**	Russia	253.27	17.44	63.74	5
**PI507730**	Russia	489.2	31.48	36.16	5

**PI507735**	Russia	377	27.61	37.14	5
**PI507757**	Russia	285.37	16.19	91.48	4
**PI507805**	Russia	157.6	11.3	29.02	4
**PI507847**	Russia	323.43	22.13	28.39	5
**PI522193**	Russia	364.55	23.59	71.66	4
**PI522196A**	Russia	303.1	20.38	32.88	4
**PI522198A**	Russia	186.75	10.55	58.14	5
**PI522200A**	Russia	324.33	20.92	56.3	4
**PI522211B**	Russia	306.2	18.57	27.62	4
**PI522212B**	Russia	276.49	20.01	33.96	5
**PI522217**	Russia	303.95	19.5	55.88	3
**PI522223**	Russia	247.05	16.6	53.66	4
**PI522226**	Russia	197.57	13.31	84.3	4
**PI522227**	Russia	341	22.59	65.75	4
**PI522229**	Russia	271.65	18.27	28.84	3
**PI522230A**	Russia	292.77	18.99	79.61	5
**PI522234**	Russia	218.53	13.41	59.42	6
**PI578336**	Russia	179.5	10.43	64.63	4
**PI578338A**	Russia	112.13	6.21	33.89	3
**PI578345**	Russia	65.28	NA	49.29	4
**PI326581**	Russia	241.95	16.55	73.04	5
**PI507723B**	Russia	396.73	25.48	48.85	5
**PI507725B**	Russia	358.67	23.99	43.1	5
**PI507727**	Russia	275.8	18.78	58.27	5
**PI507729**	Russia	412.07	27.96	51.48	4
**PI507731**	Russia	487.9	33.45	78.02	6
**PI507734**	Russia	276.9	19.53	11.62	5
**PI507736**	Russia	169.46	11.5	33.28	4

**PI507739B**	Russia	198.64	13.57	74.99	4
**PI507740**	Russia	130.75	7.95	61.13	5
**PI507742**	Russia	273.87	18.81	62.33	5
**PI507749**	Russia	252.8	15.25	60.54	5
**PI507752**	Russia	496.33	32.93	60.23	5
**PI507759**	Russia	204.87	13.59	91.78	4
**PI507760**	Russia	427.05	25.55	64.75	5
**PI507764**	Russia	223.97	14.68	61.98	4
**PI507774**	Russia	432.57	28.39	36.64	6
**PI507776**	Russia	619.93	42.17	11.27	6
**PI507777**	Russia	160.72	10.54	46.45	4
**PI507780**	Russia	156.36	9.85	33.44	4
**PI507782**	Russia	381.17	23.34	27.73	4
**PI507784**	Russia	211.67	14.61	35.78	4
**PI507787**	Russia	326.4	19.43	10.49	5
**PI507794**	Russia	405	25.45	22.03	6
**PI507798**	Russia	199.62	12.63	12.62	4
**PI507799**	Russia	348.2	22.78	36.58	5
**PI507803**	Russia	179.33	11.47	21.6	5
**PI507806A**	Russia	322.03	18.57	12.33	5
**PI507808**	Russia	200.62	13.57	19.06	4
**PI507814**	Russia	311.55	19.99	20.58	5
**PI507815**	Russia	94.14	5.31	12.84	3
**PI507818B**	Russia	284.98	19.16	70.24	4
**PI507821**	Russia	342.77	22.65	60.81	5
**PI507826**	Russia	261.93	17.26	8.32	4
**PI507830B**	Russia	303.7	20.75	48.72	4
**PI507836**	Russia	212.6	14.06	102.25	4

**PI326582A**	NA	551.7	35.2	79.64	6
**PI597471D**	NA	413.87	24.86	45.37	6
**PI639590A**	NA	345.23	17.47	69.93	5
**PI639590B**	NA	524.53	32.18	15.65	6
**PI639621**	NA	293.53	18.5	65.17	5

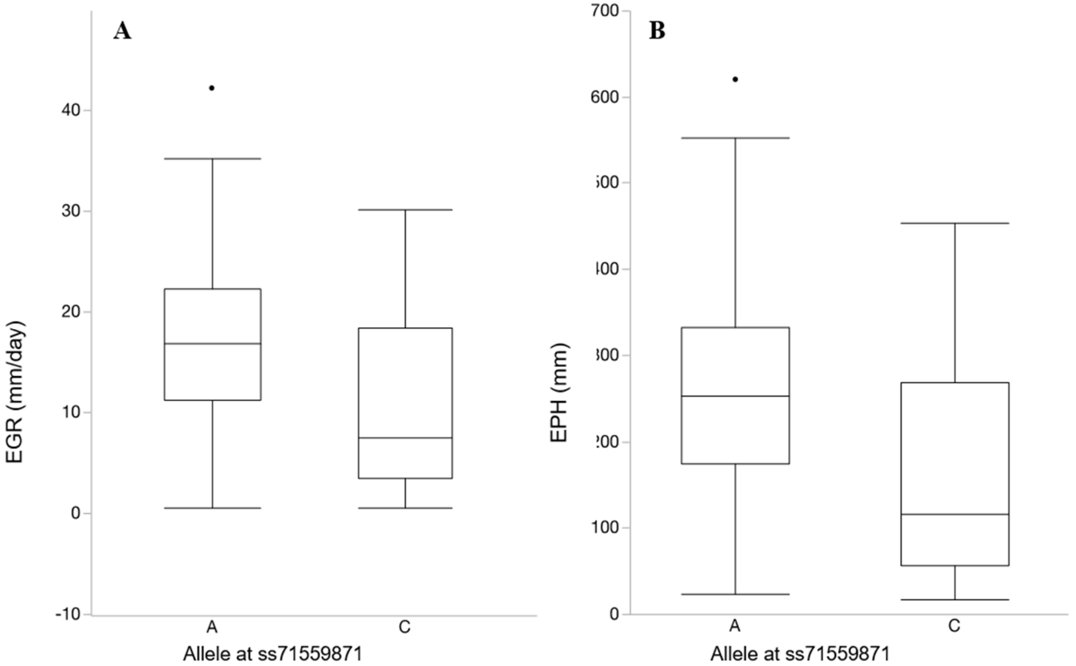

Figure S1. (A) *Glyma.07G055800.*1 allele at ss715598271 A/C comparison to EPH (mm) at marker ss715598271, p-value <0.001. **(B)** *Glyma.07G055800.*1 allele at ss715598271 A/C comparison to EGR (mm/day) at marker ss715598271, p-value <0.001.
